# Socioeconomic Inequalities in the Use of Healthcare Services: Comparison between the Roma and General Populations in Spain

**DOI:** 10.3390/ijerph15010121

**Published:** 2018-01-12

**Authors:** Daniel La Parra-Casado, Paola A. Mosquera, Carmen Vives-Cases, Miguel San Sebastian

**Affiliations:** 1 Department of Sociology 2, Alicante University, 03690 Alicante, Spain; 2 Epidemiology and Global Health, Department of Public Health and Clinical Medicine, Umeå University, 90187 Umeå, Sweden; paola.mosquera@umu.se (P.A.M.); miguel.san.sebastian@umu.se (M.S.S.); 3 Department of Community Nursing, Preventive Medicine and Public Health and History of Science, Alicante University, 03690 Alicante, Spain; Carmen.vives@ua.es; 4 CIBER of Epidemiology and Public Health, Av. Monforte de Lemos, 3-5. Pabellón 11. Planta 0, 28029 Madrid, Spain; 5 Department of Nursing I, University of the Basque Country, 48940 Bilbao, Spain

**Keywords:** ethnic groups, social class, Roma, healthcare disparities, Spain

## Abstract

This paper explores whether the principles of horizontal and vertical equity in healthcare are met by the Spanish national health system in the case of the Roma and general populations. The 2011/2012 Spanish National Health Survey (*n* = 21,650) and the 2014 National Health Survey of the Spanish Roma Population (*n* = 1167) were analyzed. Use of healthcare services was measured in terms of visits to a general practitioner (GP), visits to an emergency department, and hospitalizations. Healthcare need was measured using (a) self-rated health and (b) the reported number of chronic diseases. The Roma reported worse self-rated health and a higher prevalence of chronic diseases. A redistributive effect (increased healthcare service use among Roma and those in lower socio-economic classes) was found for hospitalizations and emergency visits. This effect was also observed in GP visits for women, but not for men. Vertical inequity was observed in the general population but not in the Roma population for GP visits. The results suggest the existence of horizontal inequity in the use of GP services (Roma women), emergency department visits (Roma and general population), and hospitalizations (Roma population) and of vertical inequity in the use of GP services among the general population.

## 1. Introduction

The Roma is the largest native ethnic minority in Europe with an estimated population of 10 to 12 million. The Roma population is characterized by rich diversity in terms of culture, socioeconomic position, citizenship, and other social characteristics both between and within European countries [1]. In the different countries where they reside, Roma people are characterized by relatively worse indicators in terms of the main social determinants of health: education levels, income, access to employment, employment conditions, place of residence, and social and institutional discrimination [2]. The Roma have been subject to more rejection and discrimination than any other group in Europe [3].

Studies show that the Roma population is among the groups with the worst indicators in terms of the social gradient in health [4,5,6,7]. This is consistent with the literature on social inequalities in health: the worse the social situation, the poorer the health indicators [8]. The gradient in health has also been observed within the Roma population as a whole by socioeconomic status [9,10,11], but not within Roma population subgroups that had a more homogeneous socioeconomic status [12].

The European Union (EU) is committed to reducing health inequalities between the general population and the Roma population. Since 2011, all member states have designed national strategies on Roma inclusion with targets for education, employment, housing, and health. The EU has also made improving access to healthcare a priority in order to promote social inclusion and equal opportunities for all [13].

The Spanish national health system is based on the principle of universal coverage, which guarantees equal access to a broad portfolio of health services to all residents in the country (including foreigners). The system does not make use of co-payments in the case of primary, specialty, or hospital care but it does require co-payments in the purchase of medicines. Some services such as dental care are covered—to a very limited extent—by the public system, and most dental services provided are private [14]. Despite the principle of universality, several studies on the Spanish healthcare system have pointed out deficiencies in terms of horizontal equity (equal access for equal health need irrespective of socio-economic status) and vertical equity (appropriately differential treatment of individuals with different needs) [15,16,17].

In terms of the Roma population’s access to healthcare, there is little evidence regarding inequalities between the Roma and the general population in Spain, but evidence exists in other countries [14,18,19,20,21]. Recent literature based on a national population-based survey in 2006 related to Roma health in Spain showed that Spanish Roma had higher levels of medication use and worse self-reported health behaviors and outcomes [22,23,24]. One of the studies—conducted only among women–also showed differences in access to mammography and cervical smear tests but no differences in access to primary healthcare, emergency, or hospitalization services [24]. It should be noted, however, that this research did not consider health needs.

The purpose of the present study was to compare socioeconomic patterns in the use of healthcare services between the Roma and the general populations in Spain, adjusted for health need, to test whether the principles of horizontal and vertical equity are met by the health system.

## 2. Materials and Methods

The data were obtained from two sources: the 2011/2012 Spanish National Health Survey, carried out by the Ministry of Health and the Office for National Statistics, and the 2013/2014 National Health Survey of the Spanish Roma Population, developed through a collaborative agreement between the Ministry of Health and a research group of Alicante University (https://www.msssi.gob.es). The latter is an adapted version of the Spanish National Health Survey that follows the same (abridged) questionnaire and question wording. Both data sources are at the Ministry of Health website and are public and accessible to researchers.

In both cases, respondents were selected through stratified, multistage sampling of the non-institutionalized population residing in Spain. Within each household, one person aged 16 years or older was selected to complete the questionnaire. Both surveys used face-to-face interviews conducted in the home of each interviewee. In the Roma survey, the interviewers were Roma (mostly women).

For the general population, study subjects were selected by means of probabilistic, multistage sampling using census data. The Office for National Statistics and the Ministry of Health have published detailed information about the methodologies of these surveys [25]. The 2013/2014 National Health Survey of the Spanish Roma followed the design used in 2006 [11] and is described in the general report published by the Ministry of Health [26]. In brief, the Roma population was sampled using estimates of Spanish Roma from a 2007 housing map. The research team corrected the map during the process of this study. A random proportional sample was obtained based on the Roma population’s distribution in Spain’s 17 autonomous communities, as well as on age group and gender. Towns were stratified by size and then randomly selected. Next, neighborhoods and areas where Roma live were identified, classified by the district’s time of construction, and randomly selected. Finally, households were chosen by systematic random routes with the final selection of cases being limited by gender and age quotas.

During the 2011/2012 survey, 21,650 people were interviewed (71% response rate) [25]. The National Health Survey of the Spanish Roma Population interviewed 1167 subjects of Roma origin with Spanish citizenship living in mainland Spain (this excludes the cities of Ceuta and Melilla in northern Africa, and the Canary and Balearic Islands). The response rate for this survey was not available [26].

Three variables were selected related to the use of healthcare services: (a) a visit to a general practitioner in the 4 weeks prior to the interview; (b) a visit to an emergency department in the last 12 months; and (c) a hospitalization in the past year.

Socioeconomic status was measured by occupational social class, which was based on pre-coded occupational categories according to the 2012 National Classification of Occupations: class I includes directors and managers with 10 or more employees and professionals with university degrees; class II includes directors and managers with fewer than 10 employees, professionals with university diplomas and other technical support professionals, and athletes and artists; class III includes intermediate occupations and self-employed workers; class IV includes supervisors and workers in skilled technical occupations; class V includes qualified workers in the primary sector (agriculture, forestry, fishing, and mining) and other semi-qualified workers; and class VI includes manual non-qualified workers [25]. These categories were used as a variable to compare the different social groups (classes), with the addition of the Spanish Roma interviewed via the Roma health survey as a seventh category.

Two variables were selected to represent the need for health services: (a) self-rated health based on the question, “In the last 12 months, how would you say your health has been?” with the response categories “very good”, “good”, “fair”, “poor” or “very poor”; and (b) the number of chronic diseases reported by the respondents during the survey. Respondents were asked whether a physician had ever told them that they suffered from hypertension, high cholesterol, diabetes, or depression (response categories: yes/no/don’t know/no answer). The replies to these four questions were grouped into a single variable with the following five categories: none, 1, 2, 3, or 4 chronic diseases. In the regression analysis, this variable was reduced to four categories due to the small number of cases in the last category.

Age and education (does not read and write, primary, secondary, and tertiary) were included in the analysis as confounding variables. Occupation and income were not included since the National Health Survey of the Spanish Roma Population did not provide comparable questions.

The two databases were merged according to the variables that were the same in both. Prevalence of the different sociodemographic and health outcome variables—stratified by gender—were obtained. Given the different age distributions of the populations, prevalence rates were measured by using directly standardized prevalence rates with the Spanish population in 2011 (last population census) as the standard (reference) population.

To assess horizontal equity, prevalence ratios and their 95% confidence intervals were calculated for the healthcare services use (dependent) and occupational social class variables, adjusted for age, education, and health need (self-rated health and number of chronic diseases). The analysis of vertical equity included the regression of severity of health need (self-rated health and the number of chronic diseases) on access to health care (visit to a general practitioner (GP), emergency service, and hospitalization) (model 1) adjusted for age and education. Interaction terms between the general/Roma populations and indicators of need (severity of self-rated health and number of chronic diseases) were also applied (model 2) to explore if there were differences in access between the two populations. Since interaction terms were significant for “a visit to GP”, stratified analyses for the general/Roma populations and gender were also conducted. Goodness of fit was assessed using the Bayesian information criterion (BIC). All statistical tests were calculated at a significance level of alpha = 0.05, and analyses were carried out using STATA 13 (StataCorp LLC, College Station, TX, USA). All estimates of regression analyses included weights to incorporate sampling design. Since differences in healthcare use by gender have been found in previous studies in Spain, all analyses were performed separately for men and women.

## 3. Results

The Roma population was younger and less educated than the general population. The Spanish Roma reported worse health in terms of self-rated health and a slightly greater number of chronic diseases (data not tabulated).

The relationship between occupational social class and the use of healthcare services is presented in Table 1. The Roma population tended to make more use of healthcare services (visits to a GP, emergency visits, hospitalizations) than the general population, both for men and women.

Among men, there was no difference by social class observed in the use of primary healthcare services after adjusting for health needs, but a positive association was found between a visit to a GP and being a Roma woman. A higher probability of emergency service use was observed for all social classes, both in men and women, with the Roma population presenting the greatest magnitude. The prevalence of hospitalizations was higher among class IV (V also in women) and in Roma as compared to class I in the general population.

Table 2 presents the results of the analysis of vertical equity for the severity of self-rated health. Those who reported more severity had more emergency visits and hospitalizations in the last 12 months (model 1) but not visits to the GP. Model 2 shows no inequalities in access according to severity between the general and Roma populations for both men and women (no significant interaction terms), for visits to emergency services and hospitalizations. However, it does show significant inequalities in terms of visits to the GP.

Table 3 presents a similar analysis but uses health need in terms of the number of chronic diseases as the outcome. As in the prior model, no vertical inequality in the use of emergency services and hospitalization was found; however, differences in access in terms of visits to the GP were observed (significant interaction term). Therefore, a stratified analysis by survey and gender was carried out for visits to the GP and health need (Table 4). The results show an inequality in access in the general population but not in the Roma population where the probability of GP visits increased with health need, both in self-rated health and in the number of chronic diseases.

## 4. Discussion

Our results show that access to healthcare in Spain is different depending on the social group in which the population is categorized. In this study, the group that made the least use of healthcare services was social class I (directors and managers with 10 or more employees, and professionals with university degrees) while the highest level of healthcare service use occurred among the Roma population. At the same time, the Roma reported worse health than the general population in Spain, indicating that the health system in Spain could be having a redistributive effect on health inequalities. This effect has different patterns depending on the type of service being analyzed.

Regarding horizontal equity, a pro-Roma distribution was observed for the three healthcare service use outcomes: GP (only for women), emergency, and hospitalization services. Emergency services (for the low and middle social classes) and hospitalizations (for the middle social class) were also more used by the general population when adjusted for health needs.

Similar results regarding GP use have been found in the scientific literature for Spain, but studies do not include a specific analysis of the Roma population [16,17] where a pro-poor horizontal equity in GP visits was reported. Despite the economic crisis, it seems that the primary healthcare system in Spain has been able to maintain equal access for the population according to their health needs. The significant result among Roma women might indicate an overuse of health services; however, it could also be a real demand not captured by the health need variables used in our study.

The pattern observed in the use of the emergency services where lower social classes (Roma included) had higher rates of service use contrasts with previous Spanish studies where no social differences were found [17]. While the severity of health conditions among these groups could partly explain this pattern, access to emergency services depends mainly on the initiative of the patient, his or her family environment, and health-seeking behavior norms. Avoidance of first level contact with primary care services could also influence this finding.

Skilled manual workers (social class IV) and the Roma population showed overuse of hospitalization services. Previous studies in Spain have found different results with some observing no inequality among social classes in terms of hospitalizations [16,17,27,28] and others a greater use by the lower social classes [28]. Interpretation of this result is not straightforward given that hospitalization depends more on doctors’ prescriptions than on patient demand. However, it could be that this effect is observed—particularly among the Roma—because of cultural conceptions of health that are not well understood by the physician or because the self-reported measures used in the study did not reflect the severity and thus hospitalization need.

Data on the Spanish Roma population contrast with data obtained in other European countries that describe unequal access to health services due to language barriers, lack of citizenship, lack of recognition of the right to health, inaccessibility of services (i.e., indirect costs, distances), acceptability factors, and discrimination [29,30,31]. Many of these barriers do not exist in the Spanish case because the Spanish Roma are Spanish citizens, they speak the language of the majority, and they have access to a system of universal public healthcare. However, other factors, mostly related to the quality of care, such as experiences of discrimination in their contact with health professionals, are still present [32,33] and require further attention.

Most studies that assess the use of healthcare have focused on horizontal equity and, as a consequence, have tended to overlook vertical equity [34]. Our study has revealed vertical inequity in access in the general population but not in the Roma population where the probability of GP visits did not increase with the severity of health need, both in self-rated health and in the number of chronic diseases. Reasons for this finding are not clear and require further exploration that is outside the scope of this paper.

Several factors should be considered when interpreting the results of this study. First, the sample used by the Spanish National Health Survey does not distinguish between Roma persons and the rest of the Spanish population. The Roma population in Spain has been estimated at 1.5% to 2.1% of the country’s total population and tends to be included in social classes IV, V, and VI. Second, the sampling method used by the Spanish National Health Survey of the Roma population consisted of selecting those areas in randomly chosen towns where a larger number of Roma people live. Thus, Roma residing in other neighborhoods, presumably those in a better socioeconomic position, were not included in the sample. As a consequence, the resulting Roma sample was homogeneous in terms of the occupational social class and it was not possible to analyze the internal heterogeneity of the Roma group (also because of sample size limitations). Additionally, probabilistic household sampling based on official registers of inhabitants cannot be applied in the case of the Roma population in Spain. For that reason, random routes were used. Therefore, the analysis assumes that all units have the same selection probability, even though participation bias could be expected. Research comparing both methods for the general population in Spain concludes that random routes and quota sampling can provide a good representation of the population distribution for some variables [35]. In this survey, the response rate was not available but reports by the field-work team described a high motivation to complete the questionnaire once a Roma household was identified because of the face-to-face interview with Roma interviewers. To ensure reliability, a standard questionnaire previously used in both populations was applied, but the extent to which the measurements can be reproduced was not possible to assess. Finally, healthcare need was measured using (a) self-rated health and (b) the reported number of chronic diseases, as is commonly done in the literature, but more accurate variables would be needed to assess vertical inequity.

## 5. Conclusions

The results of the present study suggest the existence of horizontal inequity in the use of GP services (Roma women), emergency services (Roma and general population), and hospitalizations (Roma population) and of vertical inequity in the use of GP services among the general population. Overall, the results indicate a redistributive effect of the healthcare system among the different social classes. Reasons why the general population with a higher health need are not making use of GP services requires further research.

Similarly, these results indicate that to achieve the goal of reducing health inequalities affecting the Roma population, as raised in the European Commission Framework for National Strategies for Social Inclusion, attention to the other social determinants of health (i.e., discrimination, education, labor market, and housing) should be considered in addition to the access to care.

## Figures and Tables

**Table 1:** Age-standardized prevalence (%) and prevalence ratios (with 95% CI) for the use of healthcare services by men and women according to social class.

	Men		Women	
	Prevalence	PR (95% CI) *	Prevalence	PR (95% CI) *
Visit to general practitioner (last 4 weeks)				
Social class I	37.31	1.00	38.20	1.00
Social class II	37.34	0.99 (0.86, 1.16)	40.35	0.97 (0.85, 1.13)
Social class III	38.93	1.01 (0.90, 1.14)	39.18	1.03 (0.92, 1.16)
Social class IV	39.85	1.02 (0.90, 1.15)	39.66	1.05 (0.92, 1.19)
Social class V	39.05	1.01 (0.90, 1.13)	37.86	1.05 (0.94, 1.18)
Social class VI	41.81	1.07 (0.94, 1.22)	40.40	1.08 (0.95, 1.23)
Roma	43.90	0.99 (0.86, 1.14)	55.23	1.28 (1.12, 1.46)
BIC		−73,764.21		−83,946.81
Visit to emergency services (last 12 months)				
Social class I	17.52	1.00	23.96	1.00
Social class II	22.97	1.30 (1.01, 1.67)	27.29	1.08 (0.88, 1.34)
Social class III	23.46	1.49 (1.21, 1.83)	27.66	1.15 (0.97, 1.38)
Social class IV	28.37	1.75 (1.42, 2.15)	30.67	1.33 (1.10, 1.60)
Social class V	25.16	1.52 (1.24, 1.86)	30.27	1.32 (1.11,1.57)
Social class VI	25.61	1.35 (1.08, 1.70)	31.19	1.36 (1.11, 1.63)
Roma	39.33	2.21 (1.77, 2.77)	44.20	1.98 (1.63, 2.41)
BIC		−75,047.9		−84,526.44
Hospitalization (last 12 months)				
Social class I	5.87	1.00	8.20	1.00
Social class II	8.65	1.53 (0.94, 2.50)	8.77	1.29 (0.85, 1.96)
Social class III	7.48	1.38 (0.96, 1.98)	10.60	1.37 (0.99, 1.90)
Social class IV	9.00	1.47 (1.00, 2.17)	10.56	1.51 (1.06, 2.16)
Social class V	8.80	1.32 (0.94, 1.86)	10.45	1.39 (1.01, 1.91)
Social class VI	8.82	1.29 (0.85, 1.97)	9.94	1.25 (0.88, 1.78)
Roma	12.53	1.54 (1.02, 2.31)	14.94	1.84 (1.27, 2.66)
BIC		−80,035.67		−90,508.02

* adjusted for age, education, and health needs (self-rated health and chronic diseases).

**Table 2:** Prevalence ratios (with 95% CI) for the use of healthcare services according to health need (self-rated health) for men and women.

	Men		Women	
	Model 1	Model 2	Model 1	Model 2
General practitioner (last 4 weeks)				
Self-rated health				
Very good	1.00	1.00	1.00	1.00
Good	1.00 (0.92, 1.09)	0.98 (0.91, 1.07)	0.96 (0.88, 1.04)	0.94 (0.86, 1.03)
Fair	0.98 (0.88, 1.09)	0.93 (0.83, 1.04)	1.03 (0.94, 1.13)	0.98 (0.89, 1.08)
Poor	1.09 (0.94, 1.26)	1.01 (0.86, 1.18)	1.04 (0.93, 1.17)	0.98 (0.87, 1.11)
Very poor	1.02 (0.90, 1.14)	0.87 (0.63, 1.19)	1.13 (0.94, 1.35)	0.99 (0.80, 1.23)
General vs. Roma	1.01 (0.90, 1.14)	0.58 (0.40, 0.82)	1.40 (1.27, 1.53)	1.64 (1.07, 2.54)
Very good * General/Roma		1.00		1.00
Good * General /Roma		1.41 (0.95, 2.10)		1.65 (1.07, 2.54)
Fair * General/Roma		2.39 (1.62,3.54)		2.52 (1.65, 3.86)
Poor * General/Roma		3.21 (2.12,4.89)		2.69 (1.72, 4.20)
Very poor * General/Roma		3.44 (2.00, 5.91)		3.25 (2.02, 5.22)
BIC	−73,843.43	−73,863.14	−87,876.46	−100,045.5
Emergency services (last 12 months)				
Self-rated health				
Very good	1.00	1.00	1.00	1.00
Good	1.17 (1.02, 1.35)	1.17 (1.01, 1.36)	1.35 (1.16, 1.57)	1.34 (1.14, 1.57)
Fair	2.04 (1.76, 2.36)	2.02 (1.72, 2.36)	2.16 (1.86, 2.52)	2.14 (1.82, 2.52)
Poor/	3.31 (2.83, 3.88)	3.34 (2.82, 3.97)	3.11 (2.66, 3.64)	3.12 (2.64, 3.69)
Very poor	3.65 (3.02, 4.40)	3.96 (3.20, 4.90)	3.67 (3.07, 4.38)	3.66 (3.00, 4.46)
General vs. Roma	1.38 (1.23, 1.55)	1.39 (0.95, 2.03)	1.52 (1.37, 1.68)	1.28 (0.77, 2.12)
Very good * General/Roma		1.00		1.00
Good * General/Roma		1.08 (0.70, 1.66)		1.22 (0.71, 2.09)
Fair * General /Roma		1.10 (0.72, 1.67)		1.27 (0.75, 2.16)
Poor * General/Roma		0.95 (0.61, 1.47)		1.12 (0.65, 1.92)
Very poor * General/Roma		0.78 (0.47, 1.29)		1.19 (0.69, 2.05)
BIC	−76,574.16	−76,542.47	−90,025.56	−100,508.6
Hospitalization (last 12 months)				
Self-rated health				
Very good	1.00	1.00	1.00	1.00
Good	1.98 (1.37, 2.85)	2.11 (1.43, 3.11)	0.91 (0.68,1.22)	0.91 (0.67,1.22)
Fair	6.14 (4.25, 8.87)	6.57 (4.43, 9.74)	2.27 (1.71, 3.03)	2.17 (1.61, 2.91)
Poor	11.96 (8.17, 17.50)	12.54 (8.33, 18.88)	4.34 (3.23,5.83)	4.31 (3.19, 5.83)
Very poor	17.93 (11.88, 27.07)	18.34 (11.64, 28.89)	7.01 (5.05, 9.72)	6.81 (4.83, 9.63)
General vs. Roma	1.14 (0.90, 1.46)	2.20 (0.86, 5.63)	1.36 (1.09, 1.70)	0.82 (0.26, 2.55)
Very good * General/Roma		1.00		1.00
Good * General l/Roma		0.46 (0.15, 1.37)		1.24 (0.35, 4.42)
Fair * General/Roma		0.43 (0.15, 1.21)		2.22 (0.69, 7.22)
Poor * General/Roma		0.56 (0.20, 1.59)		1.38 (0.41, 4.62)
Very poor * General/Roma		0.62 (0.22, 1.79)		1.74 (0.52, 5.83)
BIC	−81,696.03	−81,662.74	−96,180.38	−96,149.2

* Adjusted for age and education.

**Table 3:** Prevalence ratios (with 95% CI) for the use of healthcare services according to health need (comorbidity) by men and women.

	Men		Women	
	Model 1	Model 2	Model 1	Model 2
General practitioner (last 4 weeks)				
Chronic diseases				
0	1.00	1.00	1.00	1.00
1	0.99 (0.92, 1.07)	0.94 (0.87, 1.02)	1.07 (0.99, 1.15)	1.04 (0.97, 1.12)
2	0.94 (0.84, 1.04)	0.89 (0.80, 1.00)	1.04 (0.96, 1.14)	1.00 (0.91, 1.09)
3	0.91 (0.77, 1.08)	0.86 (0.72, 1.04)	1.08 (0.96, 1.21)	1.01 (0.89, 1.15)
Ethnicity	1.00 (0.89, 1.12)	0.69 (0.57, 0.82)	1.40 (1.27, 1.53)	1.10 (0.95, 1.27)
0 * General/Roma		1.00		1.00
1 * General/Roma		2.26 (1.79, 2.85)		1.35 (1.09, 1.67)
2 * General/Roma		2.34 (1.75,3.13)		1.83 (1.47, 2.27)
3 * General/Roma		2.34 (1.52, 3.59)		1.77 (1.41, 2.23)
BIC	−73,852.81	−73,886.56	−87,880.24	−87,889.71
Emergency services (last 12 months)				
Chronic diseases				
0	1.00	1.00	1.00	1.00
1	1.19 (1.07, 1.33)	1.16 (1.04, 1.30)	1.21 (1.10, 1.32)	1.18 (1.07, 1.30)
2	1.47 (1.29, 1.67)	1.44 (1.25, 1.64)	1.42 (1.27, 1.57)	1.38 (1.24, 1.55)
3	1.73 (1.46, 2.05)	1.68 (1.39, 2.02)	1.60 (1.42, 1.81)	1.57 (1.37, 1.80)
General vs. Roma	1.49 (1.31, 1.69)	1.29 (1.07, 1.55)	1.56 (1.40, 1.75)	1.34 (1.12,1.60)
0 * General/Roma		1.00		1.00
1 * General/Roma		1.35 (1.03, 1.78)		1.35 (1.05, 1.74)
2 * General/Roma		1.26 (0.92, 1.74)		1.33 (1.12, 1.60)
3 * General/Roma		1.29 (0.86, 1.92)		1.23 (0.93, 1.64)
BIC	−76,136.02	−76,114.29	−89,487.37	−89,466.58
Hospitalization (last 12 months)				
Chronic diseases				
0	1.00	1.00	1.00	1.00
1	1.93 (1.58, 2.38)	1.94 (1.57, 2.42)	1.13 (0.93,1.36)	1.08 (0.88,1.32)
2	2.07 (1.63, 2.64)	2.10 (1.62, 2.71)	1.61 (1.32, 1.96)	1.54 (1.25, 1.90)
3	3.44 (2.61, 4.52)	3.32 (2.46, 4.48)	2.27 (1.81, 2.84)	2.16 (1.69, 2.75)
General vs. Roma	1.19 (0.91, 1.54)	1.19 (0.78, 1.82)	1.39 (1.10, 1.75)	0.96 (0.64, 1.44)
0 * General/Roma		1.00		1.00
1 * General/Roma		0.93 (0.51, 1.72)		1.76 (0.98, 3.14)
2 * General /Roma		0.85 (0.39, 1.83)		1.88 (1.01, 3.49)
3 * General/Roma		1.34 (0.65, 2.75)		1.79 (1.01, 3.16)
BIC	−81,260.72	−81,234.47	−95,748.22	−95,727.16

* Adjusted for age and education.

**Table 4:** Prevalence ratios (with 95% CI) for general practitioner utilization according to health need (self-rated health and comorbidity), stratified by general/Roma population and gender.

	Men		Women	
	General *	Roma *	General *	Roma *
General practitioner (last 4 weeks)				
Self-rated health				
Very good	1.00	1.00	1.00	1.00
Good	0.99 (0.91, 1.08)	1.30 (0.88, 1.92)	0.94 (0.86, 1.03)	1.48 (0.97, 2.26)
Fair	0.93 (0.83,1.04)	1.85 (1.23, 2.78)	0.98 (0.89, 1.08)	2.17 (1.41, 3.35)
Poor	1.01 (0.86, 1.18)	2.34 (1.45, 3.76)	0.98 (0.87, 1.12)	2.22 (1.41, 3.51)
Very poor	0.87 (0.63, 1.20)	2.54 (1.60, 4.02)	1.00 (0.81, 1.23)	2.63 (1.68, 4.13)
BIC	−69,071.72	−2709.206	−82,806.44	−3430.833
Chronic diseases				
0	1.00	1.00	1.00	1.00
1	0.94 (0.87, 1.02)	1.80 (1.41, 2.29)	1.04 (0.97, 1.13)	1.25 (1.01, 1.55)
2	0.89 (0.80, 1.00)	1.76 (1.31, 2.36)	1.00 (0.91, 1.10)	1.45 (1.14, 1.86)
3	0.86 (0.72, 1.04)	1.53 (1.02, 2.30)	1.02 (0.90, 1.16)	1.38 (1.07, 1.79)
BIC	−69,087.57	−2713.882	−82,813.53	−2835.895

* Adjusted for age and education.
